# Part-time jobs and anxiety-depression disorders among medical students

**DOI:** 10.1192/j.eurpsy.2026.10797

**Published:** 2026-07-31

**Authors:** I. Kacem, A. Ghenim, A. Aloui, A. Chouchane, M. Ajmi, M. Salmanah, C. Sridi, F. Chelly, M. Maoua, M. Kahloul

**Affiliations:** 1 Occupational Medicine and Professional Pathologies Department, Farhat Hached Teaching Hospital; 2 Research Laboratory LR19SP03, University of Sousse, Faculty of Medicine of Sousse; 3 Department of anesthia and intensive care; 4 Occupational Medicine and Professional Pathologies Department, Sahloul teaching hospital, Sousse, Tunisia

## Abstract

**Introduction:**

Medical students, faced with high academic and personal expenses, may engage in part-time work. This activity offers benefits, such as fostering autonomy, improving organizational skills, and gaining professional experience. However, it can also negatively affect academic performance, mental health, and personal balance, increasing stress, anxiety, and the risk of academic disengagement.

**Objectives:**

To determine the prevalence of part-time work among students at the Faculty of Medicine of Sousse and to analyze its impact on their psychological well-being

**Methods:**

This is a cross-sectional, analytical study conducted among students at the Faculty of Medicine of Sousse during the 2024/2025 academic year. Data were collected over six months using a self-administered online questionnaire distributed via digital platforms. Anxiety and depression were assessed with the Hospital Anxiety and Depression Scale (HAD)

**Results:**

The mean age of participants was 21.6 ± 1.5 years. Among the 299 students included in the study, 22.7% (68/299) reported having a part-time job. The main types of part-time work chosen by students were manual work and small businesses (32.4%), online work (22.1%), and teaching (19.1%). Half of the students holding part-time jobs had been working for at least 2 years, and the majority (77.9%) reported being satisfied with their work. Regarding anxiety, 24.5% of students without symptoms had a part-time job compared to 16.7% of those with definite symptoms. For depression, the proportions were relatively similar across categories: 23.4% without symptoms, 23.9% with possible symptoms, and 20.3% with definite symptoms were engaged in part-time work. None of these differences were statistically significant (p = 0.489 for anxiety; p = 0.840 for depression).

**Conclusions:**

These findings suggest that, in this population, holding a part-time job does not appear to negatively impact students’ psychological well-being, although continued monitoring and support for students balancing work and academic responsibilities remain important

**Disclosure of Interest:**

None Declared

